# Supplementary figures and images for: Discovery of re-purposed drugs that slow SARS-CoV-2 replication in human cells
Source: PLoS Pathog. 2021 Sep 9;17(9):e1009840. doi: 10.1371/journal.ppat.1009840 (PMC8428568; doi:10.1371/journal.ppat.1009840)

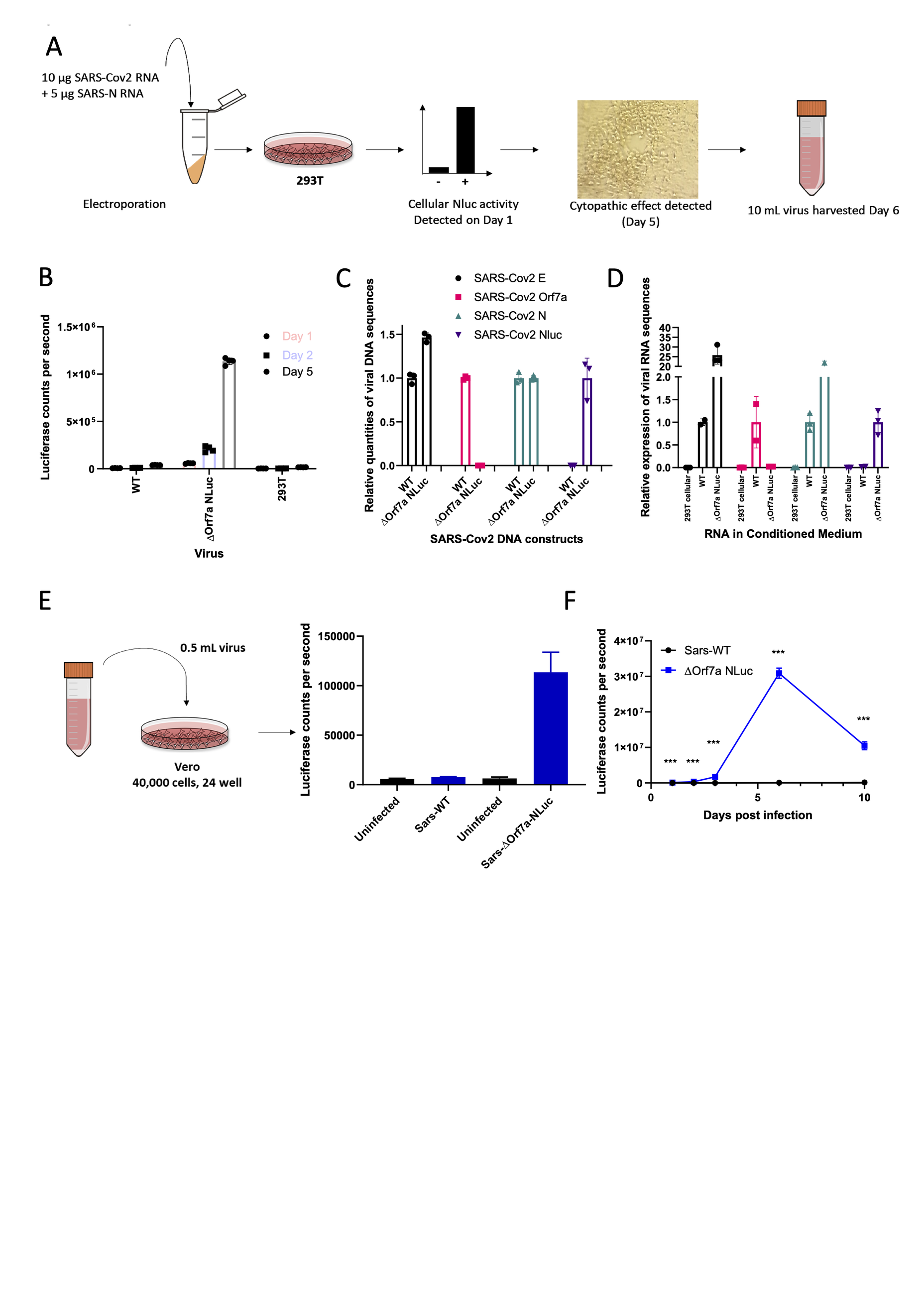

Supplement: S1 Fig — A) Schematic for the recovery of SARS-CoV-2 virus particles from DNA encoding the wild type and NLuc modified SARS-CoV-2 genome. RNA was transcribed from the synthetic DNA constructs and electroporated into 293T cells, the cells were monitored daily for NLuc activity and evidence of the cytopathic effects of virus replication. Virus containing medium was collected 6 days post electroporation. B) NLuc activity in 293T cells electroporated with wild type and NLuc modified SARS-CoV-2 RNA transcripts. C) Real-time PCR primer validation using SARS-CoV-2 encoding DNA. NLuc coding sequences were inserted in place of the Orf7a gene. Primers designed to amplify sequences within the DNA constructs confirmed primer specificity. The number of cycles for each gene were normalized to those of the WT virus, or for NLuc, to ΔOrf7a NLuc. For each DNA construct the qPCR data was also normalized to the number of cycles obtained for the N gene (N = 3 technical repeats). D) Real-time qPCR detection of viral RNAs in the medium of infected Vero cells 72 h.p.i. Vero cells were infected with SARS-CoV-2 WT (MOI 0.1) or SARS-CoV-2 ΔOrf7a-NLuc (MOI 1). E) Initial assessment of viral replication in Vero cells. Twenty-four h.p.i. NLuc activity was readily detected in SARS-CoV-2 ΔOrf7a-NLuc infected samples (N = 3 replicate samples). F) NLuc activity in Vero cells infected with wild type SARS-CoV-2 or SARS-CoV-2 ΔOrf7a-NLuc modified virus. NLuc activity was significantly elevated in SARS-CoV-2 ΔOrf7a-NLuc infected cultures across all time points, *** represents p<0.001 in a Students T-Test, N = 3 replicate samples. With prolonged culture NLuc activity within infected samples increased approximately 30-fold. (TIF) [file ppat.1009840.s001.tif]

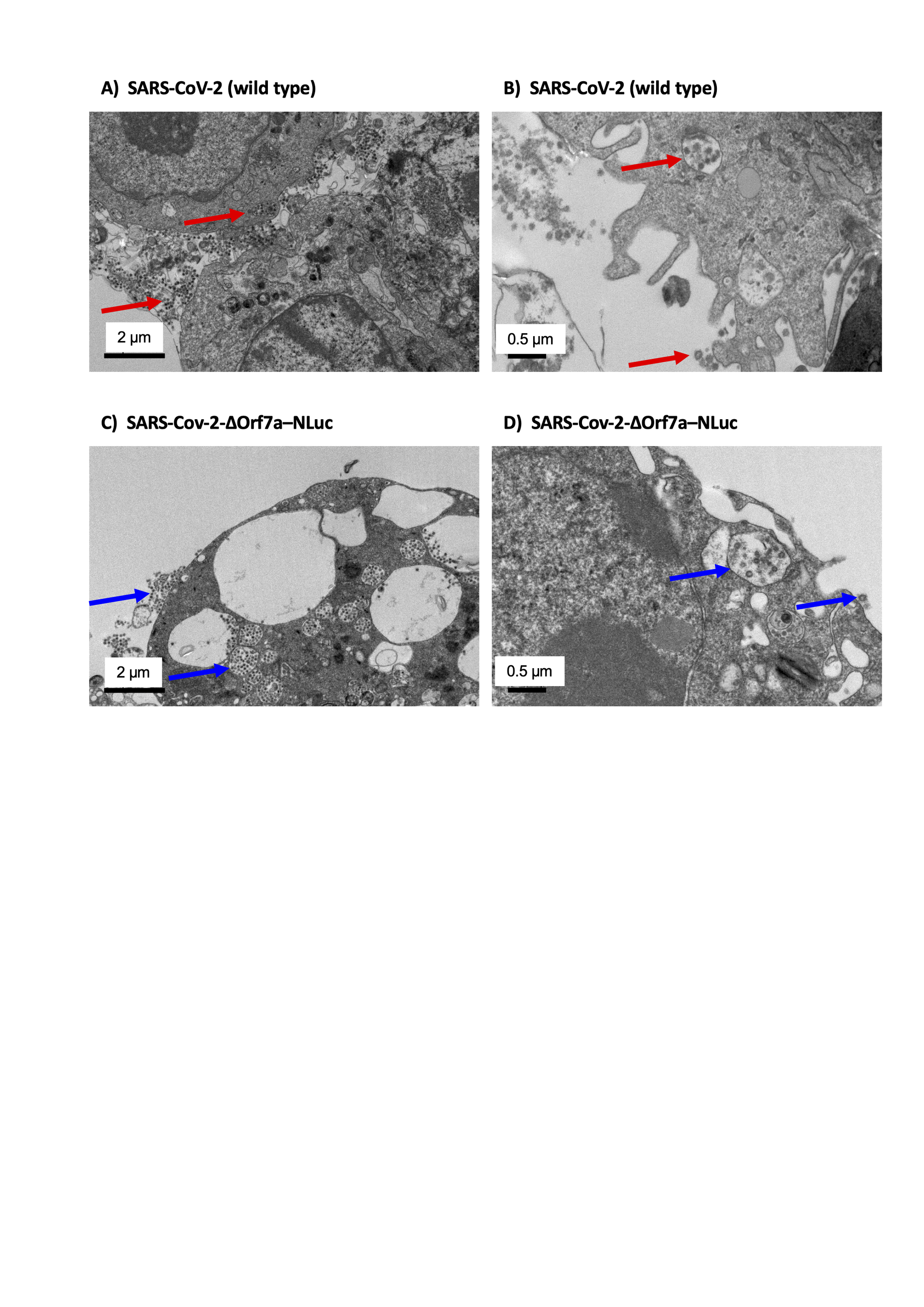

Supplement: S2 Fig — A) After 4 passages of recovered wild type SARS-CoV-2 virus particles in Vero cells, naïve Vero cells were infected and fixed 72 h.p.i. Viral particles could be observed inside and outside of Vero cells indicated by red arrows. Scale bar, 2 μm. B) Higher magnification image of an independent region of the sample in A. Scale bar, 0.5 μm. C) After 4 passages of recovered SARS-CoV-2-ΔOrf7a-NLuc virus particles in Vero cells, naïve Vero cells were infected and fixed 72 h.p.i. Viral particles could be observed inside and outside of Vero cells indicated by blue arrows. Scale bar, 2 μm. Higher magnification image of an independent region of the sample in C. Scale bar, 0.5 μm. (TIF) [file ppat.1009840.s002.tif]

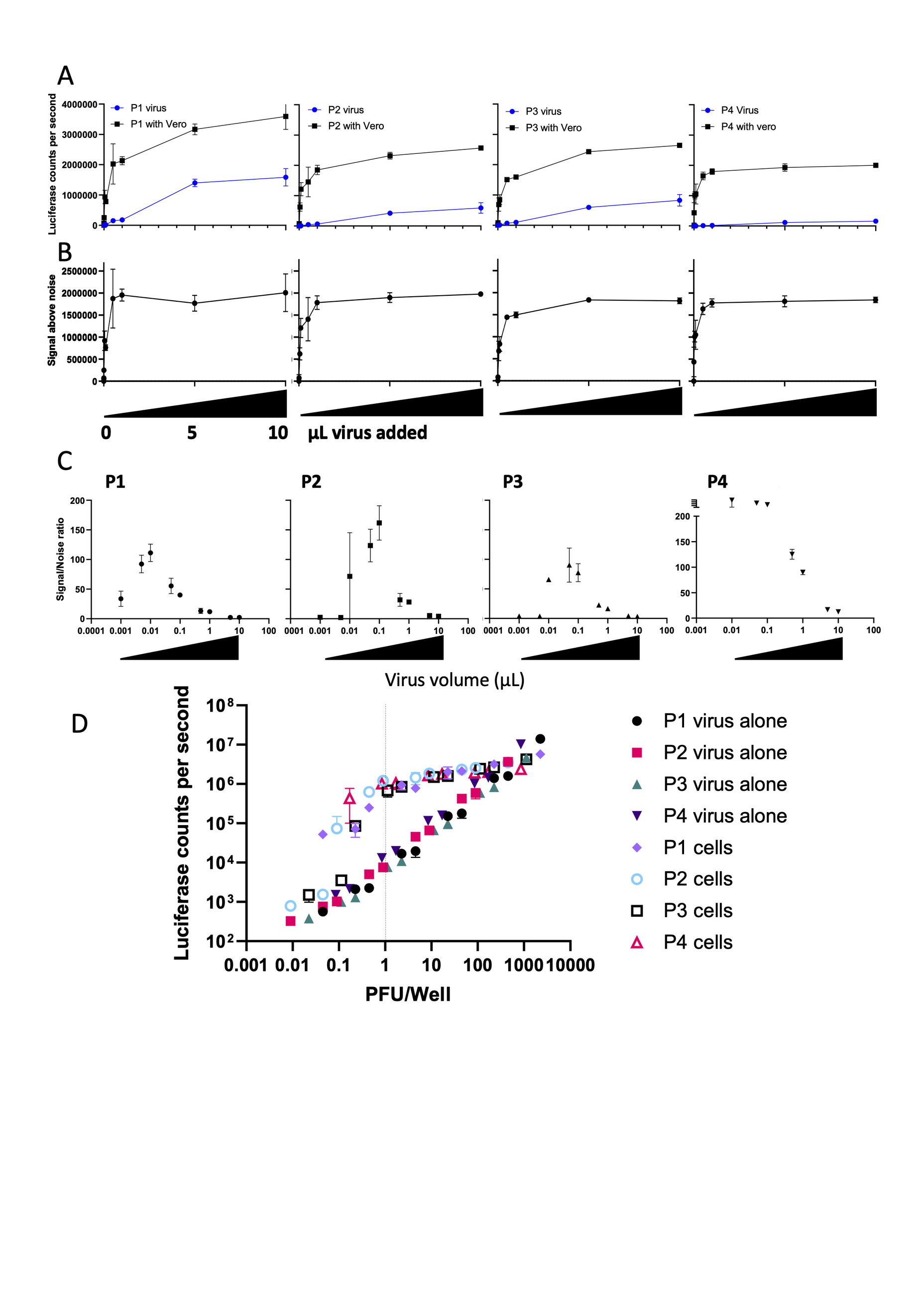

Supplement: S3 Fig — A) The plots show 5000 Vero cells infected with different volumes of virus harvested from repeat passages of the virus (P) in Vero cells. With increasing viral load, there is an increase in background as more NLuc activity is added to each well (Blue). The background signal arises when NLuc, expressed as the virus replicates, is released from infected cells that have lysed upon liberation of the virus particles. N = 2 independent experiments. Similar data were obtained with 2,000 cells and 10,000 cells as shown in S4 Fig. B) After subtracting the background noise observed in wells without cells (blue in A), the additional NLuc activity generated through viral replication showed comparable signals for each virus passage. C) The signal-to-noise ratio for each batch was used to identify the optimal conditions for virus replication for subsequent screening experiments. D) Comparison of luminescence signals for virus alone or after incubation with 5000 Vero cells. Luminescence signals are shown relative to the number of PFU for each virus stock. For all panels N = 2 independent experiments, error bars show the standard deviation of repeat measurements of both experiments. (TIF) [file ppat.1009840.s003.tif]

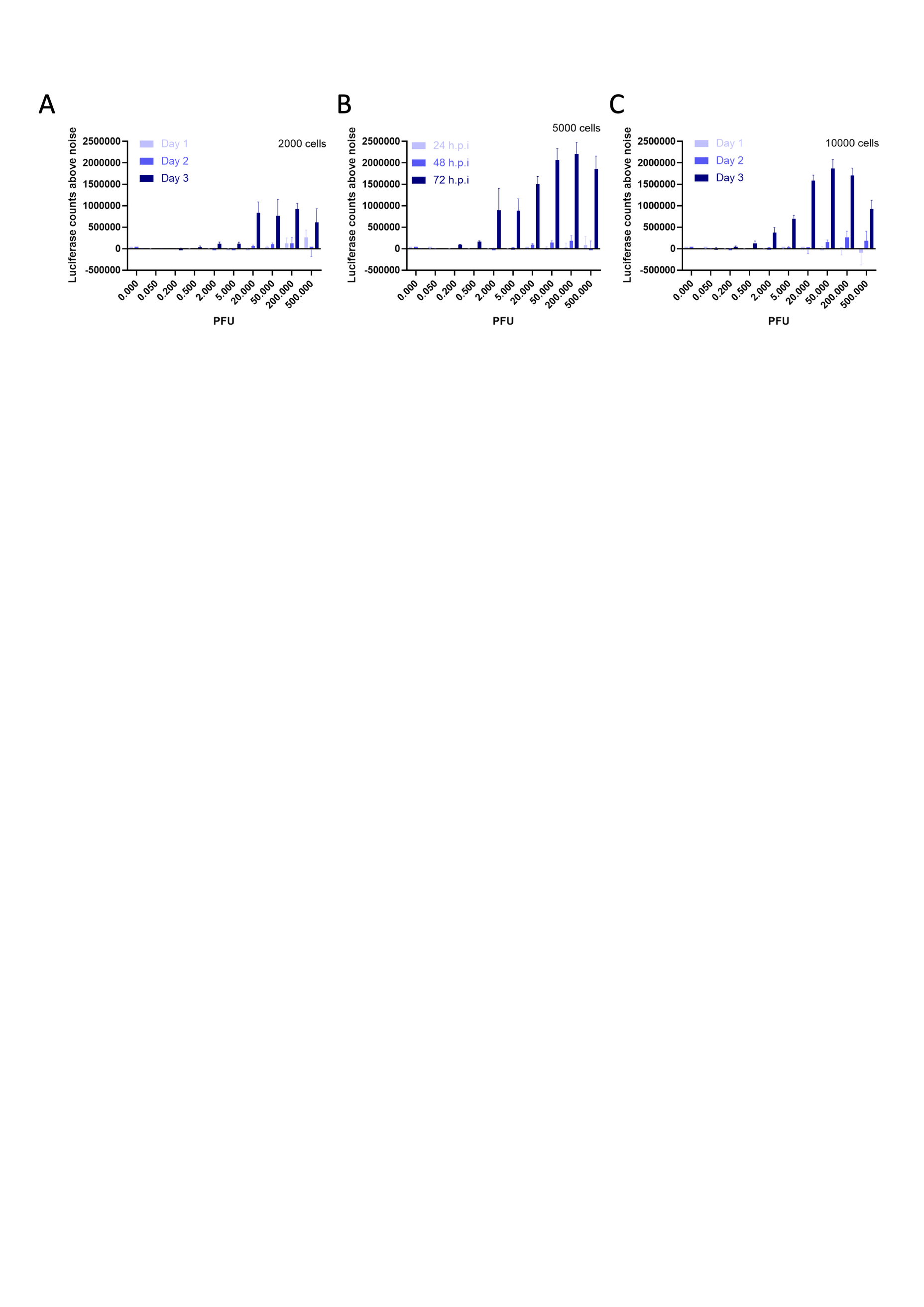

Supplement: S4 Fig — NLuc activity at different times after infection with increasing numbers of SARS-CoV-2 virus particles. Enhanced NLuc signals were observed 72 h.p.i. when either A) 2000, B) 5000 or C) 10,000 cells per well were used. Maximal signals were generated with 5000 cells per well, as seeding 10,000 cells tended to reduce NLuc activity. Five thousand cells were used for all subsequent assays. (TIF) [file ppat.1009840.s004.tif]

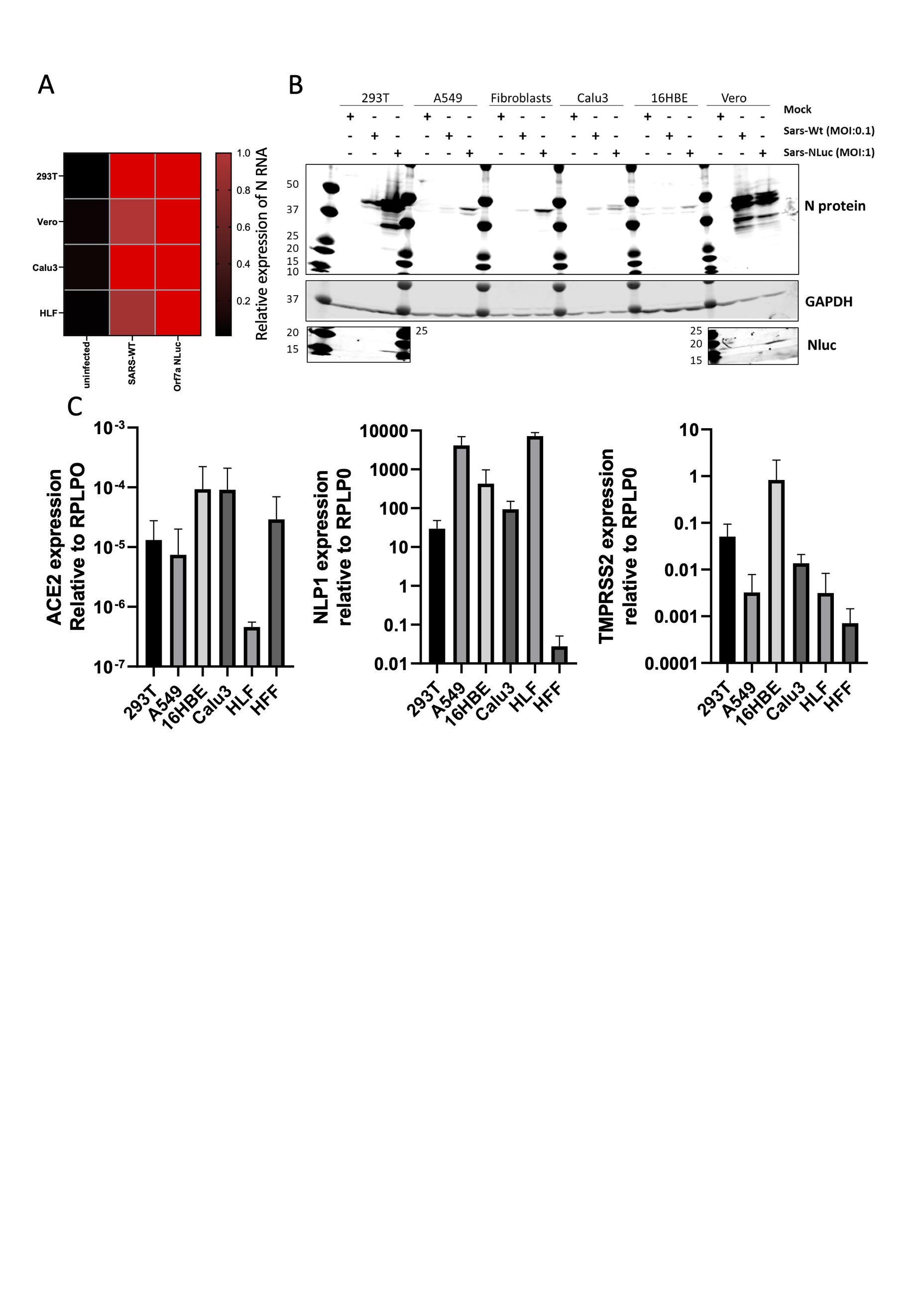

Supplement: S5 Fig — A) Real-time PCR detection of SARS-CoV-2 nucleocapsid (N) RNA in cells 72 h.p.i. Viral RNAs were readily detected in cells infected with WT or ΔOrf7a-NLuc virus particles. N = 3 independent experiments. B) Western blot detection of SARS-CoV-2 nucleocapsid (N) protein in cells 72 hours post infection. Lower levels of the N protein are detected in fibroblasts and lung epithelial cells when compared to 293T and Vero cells suggesting the virus can infect but not replicate in these cell lines. C) Real-time detection of known mediators of SARS-CoV-2 entry into cells. ACE2, NLP1 and TMPRSS2 RNA levels are shown relative to RPLP0. Infectious virus in lung cell lines but no replication. (TIF) [file ppat.1009840.s005.tif]

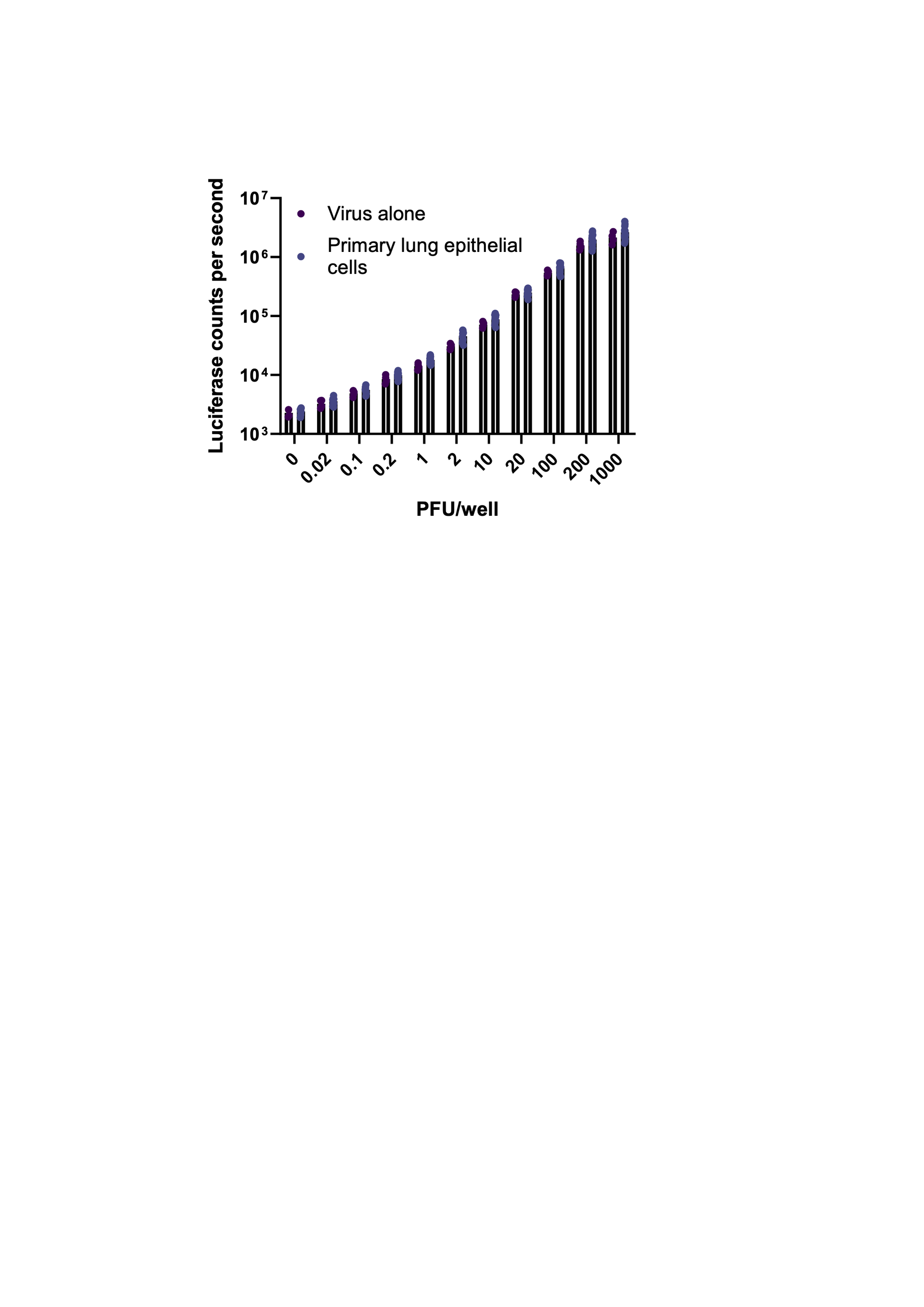

Supplement: S6 Fig — Luciferase counts per second in wells containing virus alone or virus with 5000 primary human lung epithelial cells (CC-2547, Lonza) 72 h.p.i. A small (1.25-fold) increase in signal was observed with 1000 PFU however this was not significant p>0.05, Student’s T-test and the Z’ factor in these cells was less than 0.1. (TIF) [file ppat.1009840.s006.tif]

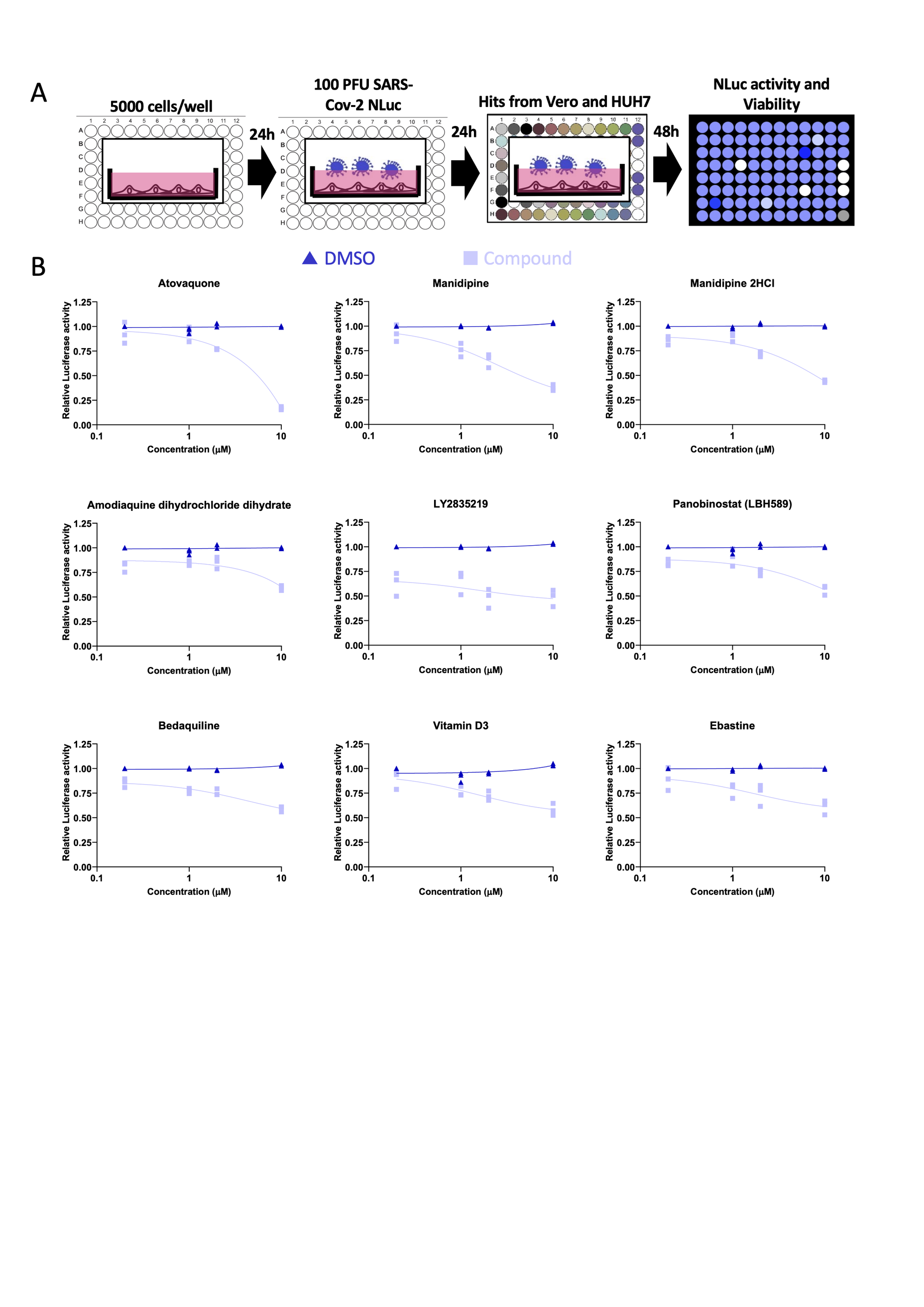

Supplement: S7 Fig — A) Schematic for assessing whether any compounds that suppressed virus replication prior to infection could also affect replication post-infection. B) Fifty compounds identified to suppress SARS-CoV-2 replication in Fig 3 were assessed to identify dose-dependent effects on SARS-CoV-2 replication with treatment progressing after infection (as outlined in A). Nine compounds were found to suppress replication in a dose dependent manner. N = 3 independent experiments, individual data points from each experiment are shown. (TIF) [file ppat.1009840.s007.tif]
