# Supplementary material for: Discovery of re-purposed drugs that slow SARS-CoV-2 replication in human cells
Source: PLoS Pathog. 2021 Sep 9;17(9):e1009840. doi: 10.1371/journal.ppat.1009840 (PMC8428568; doi:10.1371/journal.ppat.1009840)
Supplement: Attachment — Submitted filename: Response-to-reviewers-july20201.docx [file ppat.1009840.s014.docx]

| **PLoS Pathogens** |  | Prof. Karl E. Kadler PhD,  Director, Wellcome Centre for Cell-Matrix Research,  Faculty of Biology, Medicine & Health,  University of Manchester,  Michael Smith Building, Oxford Road,  Manchester M13 9PT |
| --- | --- | --- |

8^th^ July 2021

Re: PPATHOGENS-D-21-01117

Dear Editors of PLoS Pathogens,

Please find below our point by point response to reviewers.

Kind regards,

Adam Pickard and Karl Kadler

------------------------------------------------------------------------------

Reviewer #1 (Evidence, reproducibility and clarity (Required)):

**Summary:**

Pickard et al. present in the manuscript entitled "Discovery of re-purposed drugs that slow SARS-CoV-2 replication in human cells" a new screen of FDA approved drugs against SARS-CoV-2. The authors based their screen on Vero and HUH7 cell lines. The methods applied for screening including the SARS-Cov-2-ΔOrf7a-NLuc modified virus are properly designed and preformed. This is an interesting study that finds several potential drugs that might be effective as anti SARS-CoV-2 therapies. However, such experiments have been done throughout the last year and the novelty and importance of these findings are questionable.

**Regarding this point, there are several studies that have attempted to identify compounds that impact on SARS-Cov2 infection; however, these do not specifically focus on the replication of the virus (studies have used viability markers and staining of viral proteins but in these studies many of the compounds identified exert their effects by reducing virus uptake. Whilst SARS-CoV2-Nluc viruses have been developed these have been used for infection studies to measure the amount of virus taken up by cells and have not further explored how they impact on virus replication. Therefore, we feel that our study shows that a reporter virus can be used to reflect virus replication within a screening platform.**

**Major comments:**

1. Most the experiments presented are only done twice, while in the screen itself it should not be a problem, for verifying the drugs identified at least three experiments are suggested (Figure 5 and Supplemental Figure 6)

**At the time of submission there was an urgent need to make our data accessible to the scientific community. Therefore, we performed some experiments with n=2. We used n=2 to validate the screen the dose response curves also served as replicates for our screens. We have performed further repeats for figures 5 and supplemental figure 6.**

2. To strengthen the results of the screen, the wild type virus should also be tested for plaque reduction assay with these nine drugs.

**We have performed treatment of HUH7 cells and then infected these cells with WT virus. After 3 days we assessed the titre of the WT virus and its ability to replicate in human cells. These data has been added into figure 6.**

3. Identification of antivirals is important for SARS-CoV-2 and other coronaviruses, regardless of the presence or effectiveness of vaccines. I think the abstract and introduction should be written to emphasize this point (instead of trying to underestimate the vaccine effectiveness). Similarly, the authors ignore the relative failures of known antivirals (known to inhibit SARS-CoV-2 replication in vitro like Remdesavir) in clinical trials and suggest starting clinical trials with their screen results. I think that this suggestion is premature and require several more studies (including animals studies) before initiating clinical trials.

**We have re-written the abstract and introduction, and we have included information from our screens on compounds that have been trialled for effectiveness against SARS-cov2 in the clinic, and we have included new sentences in Discussion.**

**Minor comments.**

1. The errors bars are not defined throughout all the figures. I am not sure that error bars are even meaningful if experiments only done twice, I recommend showing the two results for each point.

**We have added additional repeats and defined error bars in graphs.**

2. Figure 1E and the tables especially supp tables 3 and 4? don't have legends.

**Apologies, Figure 1E and all supplementary tables now have legends.**

3. Most graphs will benefit from presenting the results in logarithmic scale (all Luc counts/ qPCRs).

**These have been amended**

4. P6 in the Generation of functional SARS-CoV-2 virus section - a reference is missing "It has been reported that this aids the recovery of replicative virus (Insert ref 3)"

**This has been amended.**

Reviewer #1 (Significance (Required)):

This is a well performed drug screen on two cell lines that identified new potential FDA approved drugs as anti-SARS-CoV-2 inhibitors. There are several studies that already been published or distributed as preprints that have done similar experiments in other cells lines including more relevant lung epithelial cells (for example PMC743673). This study does not verify the screen results by additional methods. However, in the current pandemic situation this study could be important and interesting to follow up.

I am a virologist; my expertise is in viral host interactions within infected cell.

**We were unable to identify the paper which is referred to in the reviewer’s comments. We have included data on primary lung epithelial cells, which again demonstrate low levels of replication. We have highlighted within the abstract and introduction that our screen stands out from other studies which have not examined viral replication.**

**Referee Cross-commenting**

No problem with the other comments

**Reviewer #2 (Evidence, reproducibility and clarity (Required)):**

**Summary:**

In this manuscript, the authors report on the creation of a luciferase-encoding SARS-CoV-2 (deleted for orf7a) and the use of this virus to test infectability of multiple cell lines as well as perform a drug repurposing screen in two cell lines (Vero E6 and Huh7). Of the 35 drugs that blocked the virus replication they further identify 9 drugs that have a (mild) effect on replication when administered 24 hours post infection.

**An important note here is that many studies which have identified potential therapeutics for SARS-Cov2 have performed experiments whereby cells are pre-treated with compounds prior to infection. We have been able to perform the same experiments and many of the drugs were unable to prevent replication after infection. The 9 compounds we have identified retain the ability to inhibit replication when applied post-infection. This sets our study apart from other screens that have been conducted for SARS-Cov2. A short comment about this has been included in Discussion.**

**Major comments:**

1. Figure 2: What's the difference between "Luminescence counts above noise" in Fig 2B and "Luminescence counts per second" in Figure 2C,D ? It seems like there is no difference in luminescence between 1 PFU and 100 PFU (and if anything, the bassline for 1PFU is higher, >1.5M, compared to 100 PFU where is below 1M). One would expect more luminescence in the 100 PFU experiment, as seen in Fig 2B. Also in Fig 2B it does not mention how many replicates, or what does the **** stands for.

**Thank you for the comment. The difference in “luminescence counts above noise” and “luminescence counts per second” is set out in Figure 2A. When adding more virus the baseline level should increase, as also demonstrated in Supplemental Figure 3. However, the degree of background luminescence varies between virus batches, presumably due to the degree of cell lysis in each sample. We performed the experiments in Figure 2C using virus P1 virus stocks and for Figure 2D we used P4 virus. For clarity this information has been presented as luminescence over background.**

2. The authors do not explain why deleting orf7a was needed to generate the NLuc virus. Was there a rational for this?

**Orf7a has been removed from SARS-CoV and SARS-CoV2 in order to incorporate traceable proteins such as fluorescent or bioluminescent proteins. We describe this at the start of the results section and have includeed that to include NLuc we have followed this design. “Orf7a has previously been removed in SARS-CoV and SARS-CoV-2 and yielded infectious and replicative virus particles, in order to accommodate the NLuc sequence we followed these designs (Thi Nhu Thao *et al*, 2020; Xie *et al*, 2020a; Xie *et al*, 2020b)”.**

3. Figure 5C - IC50 should be properly determined from compounds where the lowest concentration tested was still inhibitory (such as LY2835219 and panobinostat).

**We have included additional repeats of these dose response curves. However we do not feel that this would provide additional information to the reader. The aim of these figures is to demonstrate that there are dose dependent effects of these compounds on the replication of SARS-CoV2. We have now also included data relating to the wild-type virus.**

4. Supplementary tables must be provided in an excel or similar file format. The PDF version is both unreadable and does not allow other researchers to probe the dataset for their own interests.

**This has been included.**

**Minor comments:**

1. Intro: "SARS-CoV-2 infection in patients with COVID-19 can result in pulmonary distress, inflammation, and broad tissue tropism". Broad tissue tropism is not a result of infection, please rephrase.

**Patients with COVID-19 are reported to have liver and kidney damage. This could be a direct result of SARS-CoV2 infection or indirectly via the cytokine storm. Our data show that kidney and liver cells are highly susceptible to SARS-CoV2 infection and support replication in culture. We thank the reviewer for their comment and we have rephrased this statement and cited relevant literature.**

2. Fig S1D - why are the MOI different for WT (moi 0.1) and NLuc mutant (moi 1) ?

**This was used to demonstrate the lack of replication of the WT virus in lung epithelial cells, the same MOI used in Vero cells demonstrates that the levels of the nucleocapsid protein increases when compared to other cell types. We have also used an MOI of 10 for the NLuc virus to be able to detect the NLuc protein. This information has been added to the figure legend.**

3. Fig S3 - using volume of virus in ul is problematic, as it doesn't allow for proper comparison between the passages. The author would express the virus amount in PFU or MOI.

**In Figure S3 we have now included a comparison of all luminescence data for each virus passage relative to the PFU (Supplementary Figure 3D).**

4. Fig S5 - in panel A - what do the colors represent? What is 0-1?. The number of repetitions for each panel should be indicated.

**Apologies, relative expression of N RNA has now been added, N=3 for this data and this has been added to the figure legend.**

5. The "NLuc activity as a marker of virus replication" and "SARS-CoV-2 replication screen validation" are largely overlapping and should be edited.

**We have amended these sections.**

6. Methods: "Generation of functional SARS-CoV-2 virus" - the author confuse "virus" with "plasmid". They should also include the reference marked "(Insert ref 3)"

**These have been corrected in the text**

Reviewer #2 (Significance (Required)):

1. My main concern is that a very similar, if not identical, NLuc encoding virus has been reported in October 2020 (<https://www.nature.com/articles/s41467-020-19055-7#Sec9>). While the authors cite this paper, they only do so to say that "Orf7a has previously been removed in SARS-CoV and SARS-CoV-2 and yielded infectious and replicative virus particles", without mentioning this was done to generate the same NLuc carrying virus reported in their work. Thus the generation of this virus is not a "new tool" as the authors would seem to suggest.

**Whilst this is not the first use of a NLuc SARS-CoV2 virus, this is the first time that the virus has been utilised to screen for compounds that effect replication. The study mentioned does not screen nor monitor the replication of the virus. The authors do monitor the capability of the virus to infect cells but only during the first 24 hours.**

2. While drug repurposing screens have been performed, the addition validation in Vero E6 and Huh7 cells is of some interest to those working on anti-viral therapies, given that the authors change their supplementary tables to a format that can be accessible by other researchers.

**This has been amended.**

My expertise: I study virus-host interactions (not coronaviruse). In the last year I have been involved in several drug repurposing efforts against SARS-CoV-2.

**Referee Cross-commenting**

No problem with the other comments.

Dear Dr. Kadler,

Thank you for sending your manuscript entitled "Discovery of re-purposed drugs that slow SARS-CoV-2 replication in human cells" to Review Commons. We have now completed the peer review of the manuscript. Please find the full set of reports below.

Note: since the workflow of Review Commons is new, we are more than happy to schedule a call with you in case you have further questions about the content of the reviews or about the next steps, including the transfer of the reviewed manuscript to one of the affiliate journals. Please just let us know by return email and we will schedule an appointment.

To access your manuscript online and proceed to the next steps, please use the link below:

<https://reviewcommons.msubmit.net/cgi-bin/main.plex?el=A5PK6qkG6A4ZjI1I2A9ftdWuWy4VRZiHWUYoIUb0cZuwZ>

NEXT STEPS:

1. RESPONSE TO REVIEWERS: You have *four weeks* to upload to the system an *initial* response/revision plan to your reviewed manuscript (please use the link above). If you need more time to draft this document, please let us know. At this stage, a full revision is NOT expected! You have nevertheless the option to upload a preliminary revised version of the manuscript (e.g. to fix minor points or textual changes). If you prefer to do so before forwarding your manuscript to one of our affiliated journals; please ensure that all changes are visibly tracked in the file.

2. POSTING THE REVIEWS ON BIORXIV or MEDRXIV: We strongly encourage the posting of your reviews on BioRxiv and MedRxiv. You will be able to instruct Review Commons to post the reviews and your response alongside your preprint. Please follow the instructions that are provided online on the submission screen.

3. TRANSFER TO AFFILIATE JOURNALS: When you submit the response/initial revision plan (see also <https://www.reviewcommons.org/authors/#initial-revision>), you will be presented with a screen that allows you selecting the affiliate journal to which you want to submit. Please note that the full set of reviews will be transferred alongside your response and the original manuscript. If you elected to upload an initial revised manuscript, it will be transferred alongside the original version.

4. EVALUATION AT AFFILIATES: Upon transfer, the recipient journal will consider the manuscript as a formal submission and use the transferred reviews and your response/revision plan to make an initial decision. Note that while it is under consideration at an affiliate journal, the manuscript cannot be transferred to any other affiliates.

5. NEW ROUND OF TRANSFER: If the first journal declines, you will be directly informed by this journal. The manuscript will then be unlocked on the Review Commons site so that you can initiate up to two additional rounds of transfer.

Please consult our detailed Guidelines for further details on the process and policies of Review Commons at <https://reviewcommons.org/authors> . If you have any further questions, please contact me directly.

Thank you again for sending us your work. We hope you will find the reviews useful and the Review Commons helps to disseminate your peer-reviewed research faster and more efficiently.

Best wishes,
Sara Monaco --
Sara Monaco, PhD Managing Editor
Review Commons
